# UBE2S and UBE2C confer a poor prognosis to breast cancer *via* downregulation of Numb

**DOI:** 10.3389/fonc.2023.992233

**Published:** 2023-02-14

**Authors:** Yanjing Guo, Xinyu Chen, Xiaowei Zhang, Xichun Hu

**Affiliations:** ^1^ Department of Head and Neck Tumors and Neuroendocrine Tumors, Fudan University Shanghai Cancer Center, Shanghai, China; ^2^ Department of Oncology, Shanghai Medical College, Fudan University, Shanghai, China; ^3^ Department of Breast cancer and Urological Medical Oncology, Fudan University Shanghai Cancer Center, Shanghai, China; ^4^ Department of Gastrointestinal Medical Oncology, Fudan University Shanghai Cancer Center, Shanghai, China

**Keywords:** UBE2s, UBE2C, Numb, breast cancer, prognosis

## Abstract

**Purpose:**

Ubiquitin-conjugating enzymes E2S (UBE2S) and E2C (UBE2C), which mediate the biological process of ubiquitination, have been widely reported in various cancers. Numb, the cell fate determinant and tumor suppressor, was also involved in ubiquitination and proteasomal degradation. However, the interaction between UBE2S/UBE2C and Numb and their roles in the clinical outcome of breast cancer (BC) are not widely elucidated.

**Methods:**

*Oncomine*, Cancer Cell Line Encyclopedia (CCLE), the Human Protein Atlas (HPA) database, qRT-PCR, and Western blot analyses were utilized to analyze UBE2S/UBE2C and Numb expression in various cancer types and their respective normal controls, breast cancer tissues, and breast cancer cell lines. The expression of UBE2S, UBE2C, and Numb in BC patients with different ER, PR, and HER2 status, grades, stages, and survival status was compared. By Kaplan–Meier plotter, we further evaluated the prognostic value of UBE2S, UBE2C, and Numb in BC patients. We also explored the potential regulatory mechanisms underlying UBE2S/UBE2C and Numb through overexpression and knockdown experiments in BC cell lines and performed growth and colony formation assays to assess cell malignancy.

**Results:**

In this study, we showed that UBE2S and UBE2C were overexpressed while Numb was downregulated in BC, and in BC of higher grade, stage, and poor survival. Compared to hormone receptor negative (HR−) BC cell lines or tissues, HR+ BC demonstrated lower UBE2S/UBE2C and higher Numb, corresponding to better survival. We also showed that increased UBE2S/UBE2C and reduced Numb predicted poor prognosis in BC patients, as well as in ER+ BC patients. In BC cell lines, UBE2S/UBE2C overexpression decreased the level of Numb and enhanced cell malignancy, while knocking down UBE2S/UBE2C demonstrated the opposite effects.

**Conclusion:**

UBE2S and UBE2C downregulated Numb and enhanced BC malignancy. The combination of UBE2S/UBE2C and Numb could potentially serve as novel biomarkers for BC.

## 1 Introduction

The latest cancer statistics have revealed that breast cancer (BC) is the most prevalent cancer of all new diagnoses threatening women’s lives worldwide in 2020 (1, 2). The increasing global burden of breast cancer makes it urgent to seek effective biomarkers and intervention approaches for the advancement of BC treatment. Breast cancer is categorized into different molecular subtypes based on the expression of estrogen receptor (ER), progesterone receptor (PR), human epidermal growth factor receptor 2 (HER2), and antigen Ki-67, of which ER-positive (ER+) breast cancer is the most frequent molecular subtype (3, 4). Thus, finding differentially expressed genes between ER+ and ER-negative (ER−) breast cancer may contribute to understanding the pathogenesis of breast cancer and developing new druggable targets for breast cancer treatment (5).

The ubiquitination-proteasome pathway is a post-translational modification of the protein degradation system discovered in 2005 (6, 7). This biological process was mediated by E1, E2, and E3 enzymes, which carry out activation, conjugation, and ligation, respectively, and sequentially. According to recent studies, extensive progress has been made in the deregulation of the ubiquitination system, which has led to a variety of diseases, including cancer (8, 9). Ubiquitin-conjugating enzymes E2S (UBE2S) and E2C (UBE2C) are important members of the E2 family and were reported to play oncogenic roles in the tumorigenesis and progression of many cancers (10–15). In breast cancer, it has been reported that inhibition of UBE2S or UBE2C suppressed the malignant characteristics of breast cancer cells and sensitized cancer cells to radiation or drugs to enhance clinical effectiveness (11, 16–18). Besides, there was an interrelationship between UBE2S and UBE2C in regulating E3 ligase substrate modification, multiubiquitination, cell cycle progression, and drug resistance (19, 20). However, the prognostic effects as well as the collective regulatory mechanisms of both UBE2S and UBE2C in breast cancer remain to be elucidated.

Numb, widely known as a cell fate determinant and tumor suppressor in many cancers, was reported to regulate tumor suppressors, such as p53 and PTEN, and promote GLI1 oncogene degradation *via* ubiquitination (21–26). Regarding Numb expression regulation, LNX1 and LNX2, known as ligands of NUMB Protein X1 and X2, are E3 ubiquitin ligases that interact with Numb and promote its degradation (27). Besides, our previous work revealed an auto-regulatory model at the transcriptional level explaining the maintenance of a low Numb-expressing state, which promotes tumor aggressiveness in prostate cancer (28). It has been widely proven in breast cancer that Numb has a tumor-suppressive role (29, 30). In this study, we tend to explore the clinical outcome of differential expression of Numb in breast cancer and possible new regulatory mechanisms for different Numb-expressing statuses.

Given above, the expression and prognostic roles of UBE2S, UBE2C, and Numb were evaluated in breast cancer as well as in ER+ breast cancer in this research. Besides, we explored the correlation between UBE2S/UBE2C and Numb, unraveling potential regulatory mechanisms and facilitating the development of novel therapeutic strategies.

## 2 Materials and methods

### 2.1 The cBioPortal database analysis

The cBioPortal (cBio Cancer Genomics Portal) database (https://www.cbioportal.org/), which provides visualization and analysis of large-scale cancer genomics in the TCGA database, was used for coexpression analysis. In this study, the Breast Invasive Carcinoma (TCGA, Firehose Legacy) cohort was analyzed to compare the correlation between Numb and UBE2S or UBE2C. Pearson’s correlation score and Spearman score were calculated with default software parameters.

### 2.2 Oncomine database analysis

We analyzed the differential mRNA expression of UBE2S, UBE2C, and Numb in a variety of major cancer subtypes and their respective normal controls using the *Oncomine database* (www.oncomine.org). Besides, we also compared the overexpression or underexpression pattern of UBE2S, UBE2C, and Numb mRNA levels in nine independent datasets of breast cancer specimens and their matched normal tissues. Furthermore, the expression levels of UBE2S, UBE2C, and Numb were compared according to different clinical outcomes or survival statuses of breast cancer patients, providing evidence for potential predictors of prognosis.

### 2.3 Cancer Cell Line Encyclopedia analysis

The CCLE public project (https://portals.broadinstitute.org/ccle), which contains massive RNA, whole exome, and whole genome sequencing data for nearly 1,000 cancer cell lines, was applied to analyze the copy number of UBE2S, UBE2C, and Numb in certain major cancer cell lines, including breast cancer.

### 2.4 The Human Protein Atlas database analysis

Representative immunohistochemical staining of UBE2S, UBE2C, and Numb was retrieved from the Human Protein Atlas database (www.proteinatlas.org), which contains pathological information based on protein expression data from various forms of human cancer, including breast cancer, together with in-house generated immunohistochemically stained tissue section images.

### 2.5 The Kaplan-Meier plotter survival analysis

We utilized the Kaplan–Meier plotter (http://kmplot.com/analysis) web service to evaluate the correlation between prognostic values (overall survival and relapse-free survival) and investigated genes (UBE2S, UBE2C, and Numb) mRNA expression in breast cancer and the ER+ subtype based on the hazard ratios (HR) and logrank *p*-values. The high and low groups of UBE2S, UBE2C, and Numb were defined based on a 50% cutoff or the cutoff with the most significant *p*-value.

### 2.6 Cell culture and reagents

All cell lines were obtained from the American Type Culture Collection (ATCC, Manassas, VA, USA). MCF10A cells were grown in DMEM/F12 (Invitrogen, Carlsbad, CA, USA) supplemented with 10% horse serum (Invitrogen), EGF (ProSpec, Rehovot, Israel), hydrocortisone (Sigma, Beijing, China), insulin (Sigma), and 1% penicillin–streptomycin (Invitrogen). MDA-MB-231, MCF7, and T47D cells were grown in DMEM (Invitrogen). MDA-MB-468 and SK-BR-3 cells were cultured with Leibovitz’s L-15 (Gibco, Grand Island, USA) and McCoy’s 5A (Gibco), respectively. Except for MDF10A medium, all growth media were supplemented with 10% fetal bovine serum (Invitrogen), 100 Uml^−1^ penicillin, and 100 μgml^−1^ streptomycin (Invitrogen). MDA-MB-468 cells were cultured in a humidified atmosphere of 0.03% CO_2_ at 37°C while all other cell lines were cultured in the same conditions except that the CO_2_ concentration was 5%.

### 2.7 Quantitative RT-PCR

Total RNA was extracted with TRIzol reagent following the manufacturer’s instructions (Invitrogen) and proceeded to reverse transcription and real-time PCR with SYBR Premix Ex Taq (TaKaRa, Beijing, China). The expression levels of UBE2S, UBE2C, and Numb were normalized to ACTIN and calculated using the 2^−ΔΔCt^ method. The primer sequences (5’–3’):

*UBE2S*-forward: CCGACACGTACTGCTGACC;

*UBE2S*-reverse: GCCGCATACTCCTCGTAGTTC;

*UBE2C*-forward: AGTGGCTACCCTTACAATGCG;

*UBE2C*-reverse: TTACCCTGGGTGTCCACGTT;

*NUMB*-forward: TCAGCAGATGGACTCAGAGTT;

*NUMB*-reverse: AGGCTCTATCAAAGTTCCTGTCT;

*ACTIN*-forward: GCACAGAGCCTCGCCTT;

*ACTIN*-reverse: GTTGTCGACGACGAGCG.

### 2.8 Western blot analysis

MCF10A protein was extracted using M-PER containing an EDTA-free Halt Protease Inhibitor Cocktail (Thermo Fisher Scientific, Massachusetts, USA), while proteins from all other cell lines were extracted using RIPA lysis buffer. Protein transfer was performed on a nitrocellulose membrane (Bio-Rad Laboratories, California, USA). Primary antibodies include: UBE2S (11878; Cell Signaling Technology, Massachusetts, USA; 1:1,000), UBE2C (14234; Cell Signaling Technology; 1:1,000), Numb (sc-136554; Santa Cruz Biotechnology, Texas, USA; 1:200), and ACTIN (3700; Cell Signaling Technology; 1:1,000). Protein was visualized with the ECL Western Blotting Detection System (PerkinElmer, USA), and a densitometry value was determined in terms of pixel intensity by ImageJ.

### 2.9 Transient overexpression and knockdown

MCF7 and MDA-MB-231 cells were seeded in 6-well plates and cultured to 70%–80%. For transient overexpression (OE), cells were transfected with a constructed plasmid (pcDNA3.1, Thermo Fisher Scientific) overexpressing UBE2S/UBE2C or a negative control using Lipofectamine 3000 (Invitrogen). Gene sequences were obtained from the Ensembl human genome assembly (Genome Reference Consortium GRCh38). For transient knockdown, cells were transfected with small interfering RNAs (siRNAs) targeting UBE2S/UBE2C or a negative control (Shanghai Genechem Co., Ltd., Shanghai, China):

si-UBE2S-1: GAUCCUGCUUUGUGCUAAAGA;

si-UBE2S-2: GAAUCAGACAACCUUGUCAAA;

si-UBE2C-1: UGAAAAGGUUGUCUGAUUCAG;

si-UBE2C-2: GGAAAGACAAGGGAAGAAACC.

After 4-hour incubation, the medium was replaced with DMEM containing 10% fetal bovine serum. Transfection efficiency was determined 48 h later using RT-qPCR and Western blotting.

### 2.10 Colony formation assay

MCF7 and MDA-MB-231 cells were seeded in 6-well plates at a concentration of 1,000 cells per well and cultured for 10–14 days. Colonies were fixed and stained using 0.2% crystal violet.

### 2.11 Cell counting kit-8 assay

For the CCK-8 cell proliferation assay, MCF7 and MDA-MB-231 cells were seeded in 96-well plates at a concentration of 3,000 cells per well. The absorbance at 450 nm was determined at the indicated time points using a microplate reader two hours after adding 10 μl of CCK-8 solution (HY-K0301; MedChemExpress, China) to each well.

### 2.12 Statistical analysis

In this study, data obtained from the *Oncomine* database were presented using Prism GraphPad software (LaJolla) and analyzed using a Student’s *t*-test. A statistical difference was determined two-sidedly with *P*-values less than 0.05, which are indicated with the following asterisks: *P <0.05, **P <0.01, **P <0.001, ****P <0.0001. For the rest of the study’s data, statistical analyses were calculated with default software parameters. All experiments were performed in three biological replicates.

## 3 Results

### 3.1 UBE2S and UBE2C are overexpressed while Numb is downregulated in breast cancer

**Figure 1:**
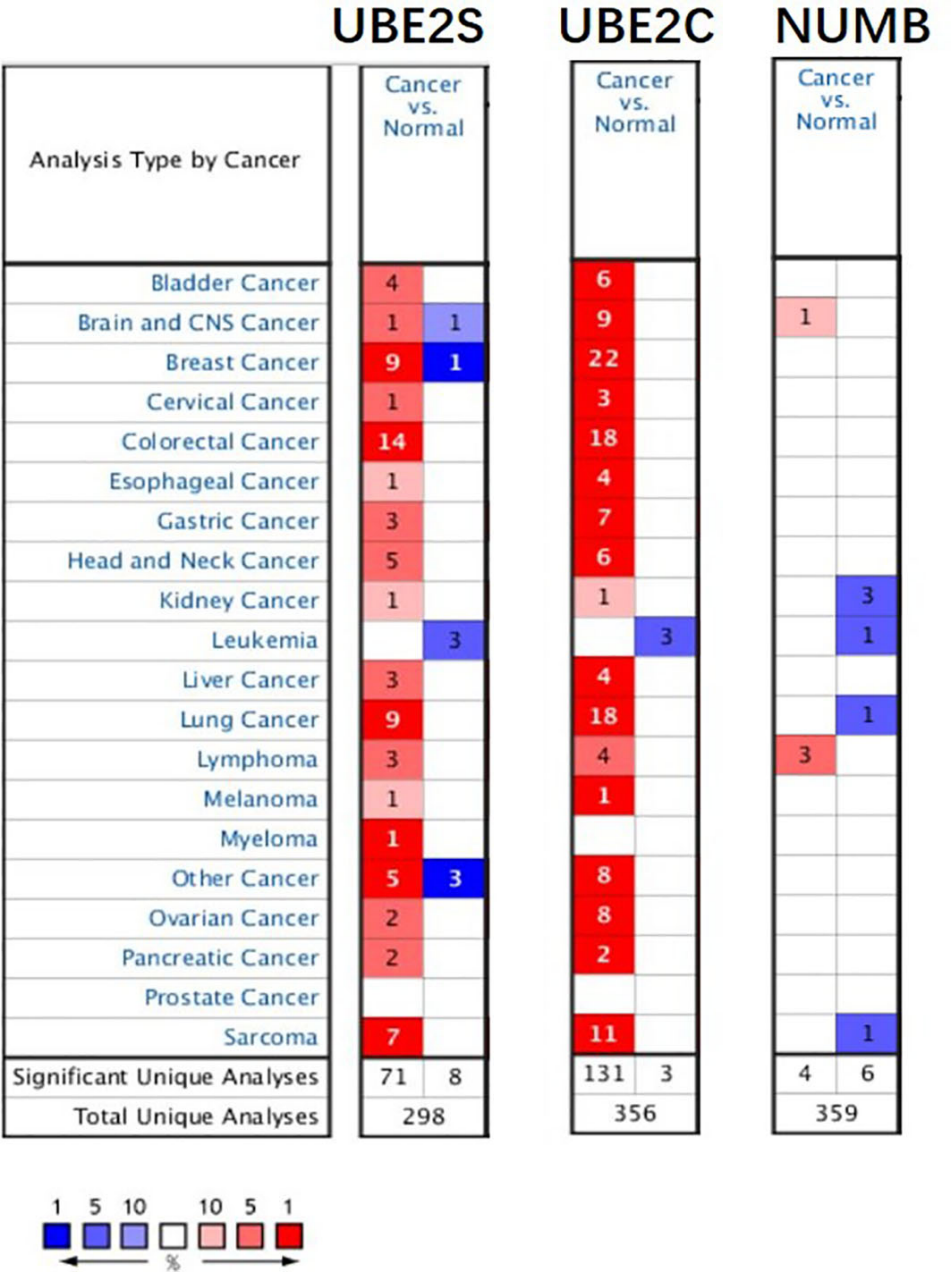
The expression of UBE2S, UBE2C, and Numb in different types of tumors (*Oncomine* database). In the diagram, the threshold parameters were set as follows: p-value of 0.01, fold change of 2, and gene rank of 10% for the cell color analysis within the cell. In each form, the number represents the number of analyses that meet the threshold across various malignancies.

**Figure 2:**
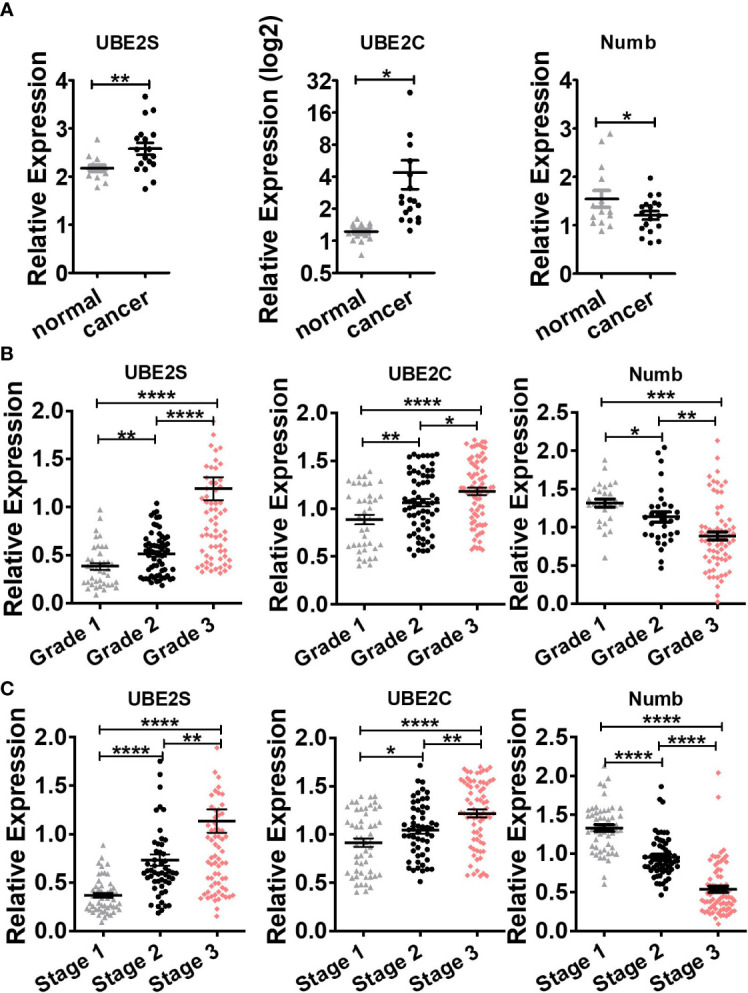
Comparison of UBE2S, UBE2C, and Numb between **(A)** normal breast tissues and breast tumors, as well as breast tumors of various **(B)** grades and **(C)** stages. **(A)** UBE2S and UBE2C are upregulated, while Numb is downregulated in breast tumors (*n* = 14) compared with normal breast tissues (*n* = 18) (*Oncomine* database of Ma breast, 2009). normal, normal breast tissues; cancer, breast cancer tissues). **(B)** With the increase in breast tumor grade (grade 1 *n* = 39, grade 2 *n* = 63, grade 3 *n* = 76), UBE2S and UBE2C show elevated expression while Numb exhibits decreased level. **(C)** With the increase in breast tumor stage (stage 1 *n* = 52, stage 2 *n* = 54, stage 3 *n* = 76), UBE2S and UBE2C show elevated expression while Numb expression decreases. A Student’s *t*-test was used for the statistical analysis. *, *P <*0.05; **, *P <*0.01; ***, *P <*0.001; ****, *P <*0.0001.

**Figure 3:**
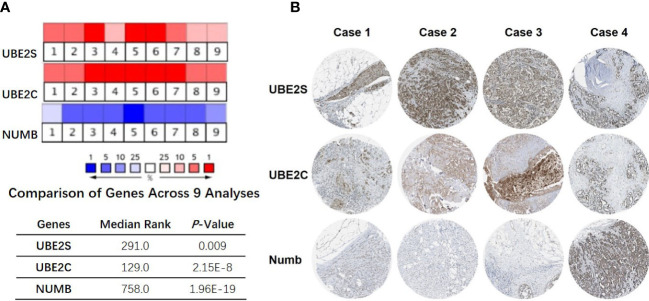
Correlation of UBE2S, UBE2C, and Numb expression. **(A)** The mRNA levels in breast cancer samples and corresponding normal controls across nine analyses (Oncomine database). The median rank and p-value for the genes across each of the analyses are shown in the graph. **(B)** Representative images of immunohistochemical staining of UBE2S, UBE2C, and Numb in four groups of breast cancer tissues, with each case from the same patients. Breast cancer tissues with medium or high protein staining of UBE2S and UBE2C have negative or weak staining of Numb protein (Cases 1–3) whereas tissues with lower levels of UBE2S and UBE2C protein expression display strong staining of Numb protein (Case 4). Data were obtained from the Human Protein Atlas (HPA) database.

To explore the expression and potential clinical significance of UBE2S, UBE2C, and Numb in breast cancer, first we analyzed the *Oncomine* microarray datasets to determine the mRNA levels of UBE2S, UBE2C, and Numb in a variety of malignant tumor types, including breast cancer. As shown in **Figure 1**, the expression of UBE2S and UBE2C was generally higher and Numb expression was lower in various cancer types than their respective normal controls, especially in breast cancer, although no obvious differential expression of Numb was shown in certain cancers. Besides, we resorted to the *Oncomine* datasets regarding breast cancer and found that there was evidently higher mRNA expression of UBE2S and UBE2C but lower Numb expression in breast cancer samples than their respective normal controls (**Figure 2A**). Next, we found that the expression of UBE2S and UBE2C was upregulated while Numb was downregulated in breast cancer tissues of higher histological grades and pathological stages (**Figures 2B, C** **)**. More specifically, the comparison of UBE2S, UBE2C, and Numb mRNA expression across nine independent analyses demonstrated that UBE2S and UBE2C were markedly elevated and Numb was significantly reduced in breast cancer (**Figure 3A**). To identify the differential expression of UBE2S, UBE2C, and Numb in breast cancer tissues at the protein level, we resorted to the Human Protein Atlas (HPA) database. Representative images of immunohistochemical staining of UBE2S, UBE2C, and Numb in breast cancer tissues from the same patients are shown in **Figure 3B**. We found that breast cancer tissues with medium or high protein staining of UBE2S and UBE2C had negative or weak staining of Numb protein (**Figure 3B**, Cases 1–3>), whereas the lower protein levels of UBE2S and UBE2C displayed strong staining of Numb protein (**Figure 3B**, Case 4). Consistently, we found out that the copy numbers of UBE2S and UBE2C were significantly upregulated in breast cancer cell lines and rank relatively high among various cancer cell types through CCLE analysis, while the Numb copy number was relatively low in breast cancer cell lines (**Figure S1**). Taken together, these results revealed an overexpression of both UBE2S and UBE2C and a downregulation of Numb in breast cancer, indicating the oncogenic effects of UBE2S and UBE2C and the tumor suppressive role of Numb in breast cancer.

### 3.2 UBE2S and UBE2C are downregulated while Numb is upregulated in ER+ BC compared with ER− BC

**Figure 4:**
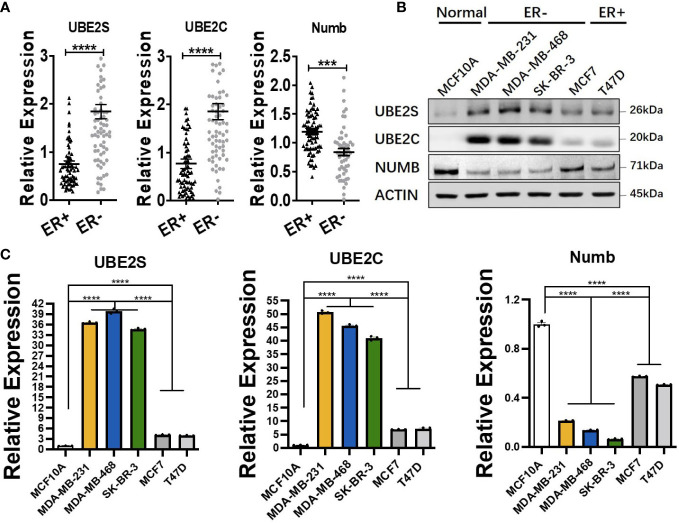
Comparison of UBE2S, UBE2C, and Numb between ER+ and ER− breast tumors. **(A)** The mRNA levels in ER+ and ER− breast tumors (ER+ *n* = 70, ER− *n* = 74). ER− breast cancer shows higher levels of UBE2S and UBE2C, as well as lower Numb expression. **(B)** Western blot for protein levels and **(C)** qRT-PCR for mRNA levels in breast cell lines. One normal breast epithelial cell line (MCF10A), three ER− breast cancer cell lines (MDA-MB-231, MDA-MB-468, and SK-BR-3), and two ER+ breast cancer cell lines (MCF7 and T47D) a re adopted. Breast cancer cell lines demonstrate higher UBE2S and UBE2C and lower Numb than normal controls. ER− breast cancer cell lines show higher UBE2S and UBE2C, as well as lower Numb expression, than ER+ breast cancer cell lines. Student’s *t*-test was used for the statistical analysis. ***, *P <*0.001; ****, *P <*0.0001.

ER+ breast cancer is the most common pathological subtype and accounts for over 70% of breast cancer (31). As the most prevalent breast cancer subtype, ER+ breast cancer was correlated with lower tumor grade and lower metastatic rate of lymph nodes and better survival (32, 33). To explore the functional role of UBE2S, UBE2C, and Numb in ER+ breast cancer, we compared the mRNA levels of the three genes in ER+ and ER− breast cancer through *Oncomine* dataset analysis. As demonstrated in **Figure 4A**, compared with ER+ breast cancer, the mRNA levels of UBE2S and UBE2C were higher, while NUMB was reduced in ER− breast cancer. To verify the above findings in breast cell lines, we adopted one normal breast epithelial cell line (MCF10A), three ER− breast cancer cell lines (MDA-MB-231, MDA-MB-468, and SK-BR-3), and two ER+ breast cancer cell lines (MCF7 and T47D). Both qRT-PCR and western blot confirmed increased UBE2S and UBE2C, as well as decreased Numb expression, in breast cancer cell lines compared to MCF10A. Compared with ER+ breast cancer cell lines, ER− cells demonstrated higher UBE2S and UBE2C alongside lower Numb, also in line with transcriptomic results (**Figures 4B, C** **)**. It is widely known that breast cancer is classified into different subtypes according to ER, PR (progesterone receptor), and HER2 (human epidermal growth factor receptor 2) status. Apart from ER+ and ER− breast cancers, we also compared the expression of UBE2S, UBE2C, and Numb in PR positive (PR+) and PR negative (PR−) breast cancers as well as in HER2 positive (HER2+) and HER2 negative (HER2−) breast cancers. As shown in **Figure S2A**, compared with PR+ breast cancer, the mRNA levels of UBE2S and UBE2C were higher, while NUMB was lower in PR− breast cancer. However, no statistical significance of UBE2S, UBE2C, or Numb expression was observed between HER2+ and HER2− breast cancer (**Figure S2B**). These data indicate that the regulation between UBE2S/UBE2C and Numb plays a more critical role in hormone receptor-positive breast cancer than in HER2+ breast cancer.

### 3.3 UBE2S and UBE2C are worse prognosis predictors in breast cancer and ER positive subtype patients in contrast to Numb

**Figure 5:**
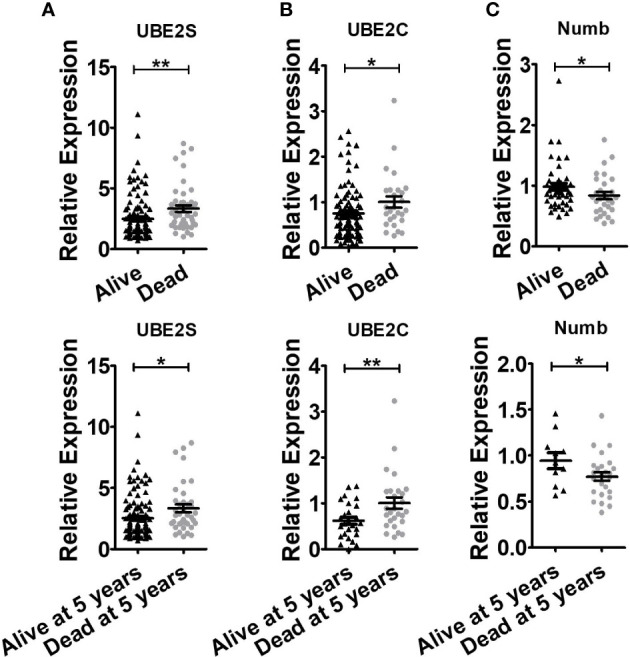
Breast cancer patients with shorter lifespans have higher levels of UBE2S and UBE2C and lower expression of Numb. **(A)** UBE2S mRNA expression in the *Oncomine* database of Pawitan Breast grouped by overall survival status (Up, Alive n = 113, Dead n = 46) and survival status at 5 years (Bottom, Alive at 5 years n = 121, Dead at 5 years n = 38). **(B)** UBE2C mRNA expression in *Oncomine* database of Esserman Breast grouped by overall survival status (Up, Alive n = 98, Dead n = 27) and survival status at 5 years (Bottom, Alive at 5 years n = 27, Dead at 5 years n = 27). **(C)** Numb mRNA expression in the *Oncomine* database of Sorlie Breast grouped by overall survival status (Up, Alive n = 46, Dead n = 30) and survival status at 5 years (Bottom, Alive at 5 years n = 11, Dead at 5 years n = 25). (*t* test was used for the statistical analysis. *, *P <*0.05; **, *P <*0.01).

**Figure 6:**
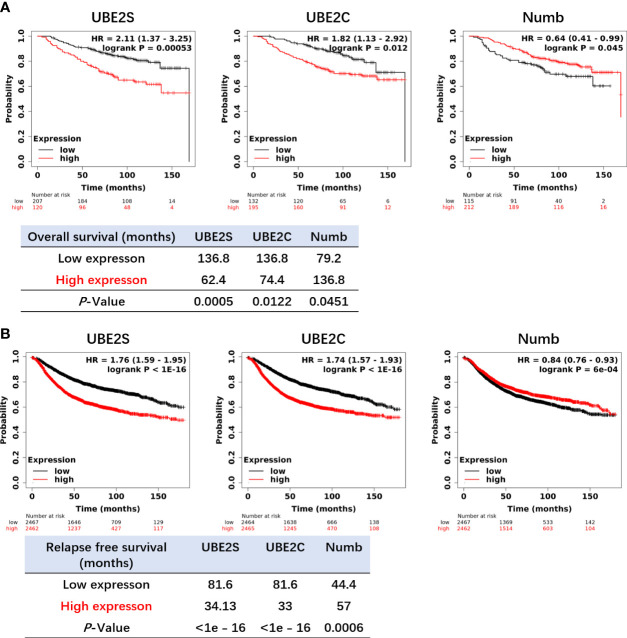
Higher levels of UBE2S and UBE2C and a lower level of NUMB correlate with shorter OS **(A)** and RFS **(B)** in breast cancer patients by the Kaplan–Meier plotter survival analysis. OS, Overall Survival. RFS, Relapse-Free Survival. The *p*-value and survival time were indicated in the respective graphs.

**Figure 7:**
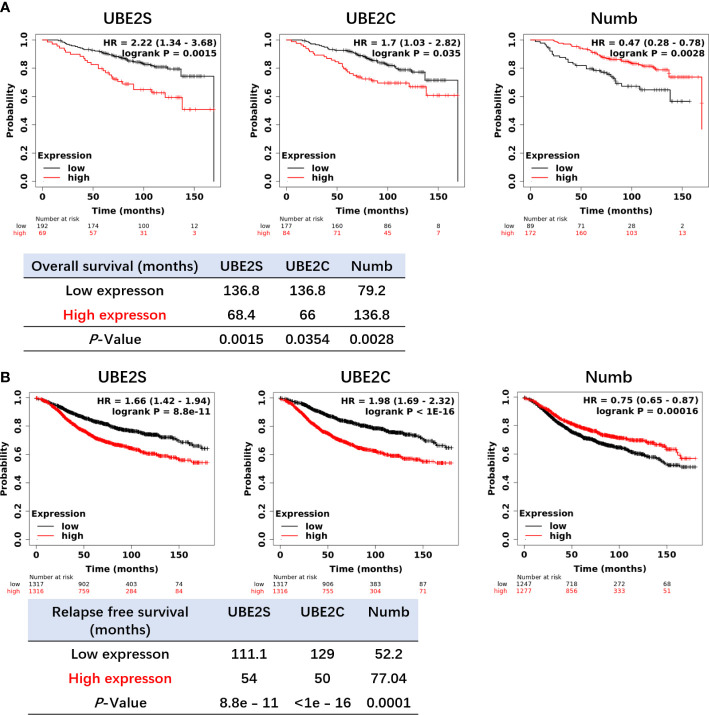
Increased expression of UBE2S and UBE2C and reduced expression of Numb are related to shorter OS **(A)** and RFS **(B)** in ER+ breast cancer patients by the Kaplan–Meier plotter survival analysis. The *p*-value and survival time were indicated in the respective graphs.

**Figure 8:**
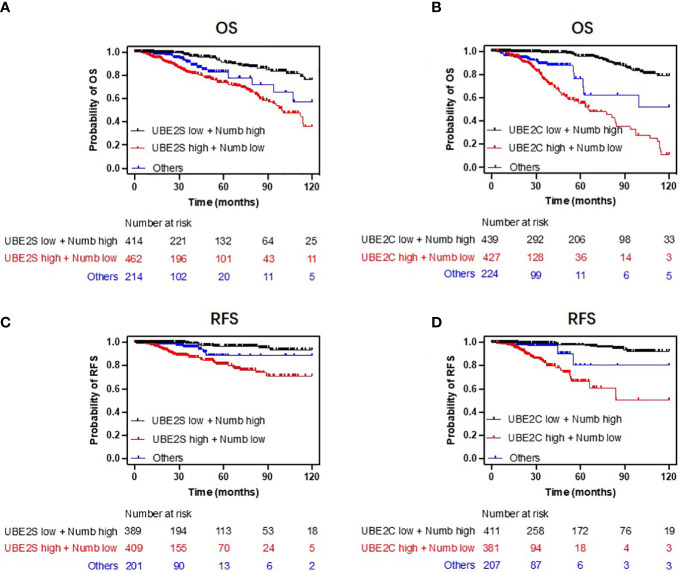
Combining UBE2S or UBE2C with Numb predicts breast cancer patients’ survival. Patients with low UBE2S or UBE2C levels and high Numb expression demonstrated higher OS **(A, B)** and RFS **(C, D)** probabilities compared to those with high UBE2S or UBE2C levels and low Numb expression. Univariate regression analyses are presented in **Supplementary Table 1**.

By using *Oncomine* data analysis and Kaplan–Meier plotter survival analysis, we further analyzed the prognostic effects of UBE2S, UBE2C, and Numb in breast cancer patients. In **Figures 5A, B**, it revealed that the mRNA levels of UBE2S and UBE2C were lower in breast cancer patients who had a longer lifespan than those with a shorter lifespan, grouped by overall survival status or survival status at 5 years (alive or dead). On the contrary, patients with a longer lifetime had higher Numb mRNA expression **(** **Figure 5C** **)**. In other words, high levels of UBE2S and UBE2C and decreased Numb expression were associated with a shorter lifespan in breast cancer patients. Next, we evaluated the impact of differential mRNA expression of UBE2S, UBE2C, and Numb on the clinical survival of breast cancer patients. We found that higher levels of UBE2S and UBE2C and lower expression of Numb were correlated with shorter overall survival (OS) and relapse-free survival (RFS) in breast cancer patients (**Figure 6**). As mentioned above, the expression of UBE2S and UBE2C was decreased and Numb was increased in ER+ breast cancer; we thus explored the prognostic values of UBE2S, UBE2C, and Numb in ER+ breast cancer patients. As demonstrated in **Figure 7**, increased expression of UBE2S and UBE2C predicted worse OS and RFS, while increased Numb expression was related to a better outcome in ER+ breast cancer patients. In ER− breast cancer patients, UBE2S, UBE2C, and Numb did not show a correlation with OS or RFS (**Figure S3**). To further clarify the negative correlation between UBE2S/UBE2C and Numb in breast cancer prognosis, we compared the OS and RFS in breast cancer patients divided into three groups: one with higher UBE2S or UBE2C and lower Numb expression (UBE2S or UBE2C high + Numb low), one with lower UBE2S or UBE2C and higher Numb expression (UBE2S or UBE2C low + Numb high), and the rest were categorized into the third group. As indicated in **Figure 8** and **Supplementary Table 1**, UBE2S or UBE2C high + Numb low patients had notably shorter OS and RFS compared with patients with UBE2S or UBE2C low + Numb high patients. Taken together, UBE2S and UBE2C were worse prognosis predictors in breast cancer patients as well as in ER+ patients, while Numb showed the opposite prognostic effect.

### 3.4 Numb is negatively correlated with UBE2S and UBE2C in breast cancer and UBE2S or UBE2C inhibit Numb expression

**Figure 9:**
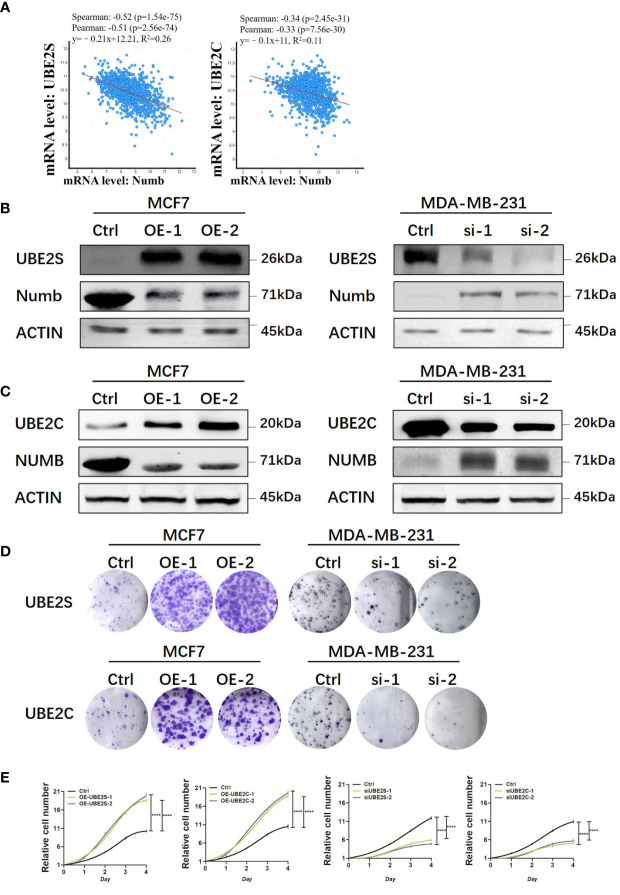
UBE2S and UBE2C inhibit Numb expression and increase colony formation and cell growth. **(A)** Numb has a negative correlation with UBE2S and UBE2C at the mRNA level in breast cancer tissues by cBioPortal database analysis. TCGA, Firehose Legacy, 1,101patients/1,108 samples. The Pearson’s correlation score, Spearman score, and *p*-value were indicated in the respective graphs. UBE2S **(B)** and UBE2C **(C)** are overexpressed (OE) in MCF7 cells and knocked down with small interfering RNA (si) in MDA-MB-231 cells, and the Numb protein level is determined through western blot. β-actin was used as a loading control. Colony formation **(D)** and the CCK-8 growth assay **(E)** are performed in these cells. Each assay was repeated at least three times.

As indicated above, UBE2S and UBE2C predicted a poorer prognosis while Numb demonstrated opposite effects, so the correlation between Numb and UBE2S/UBE2C was further investigated in the cBioPortal database to unravel potential regulatory mechanisms. As shown in **Supplementary Table 2**, there were 6,431 negatively correlated genes with Numb that were statistically significant (*P <*0.05). Among these genes, UBE2S ranked first and UBE2C ranked 526th based on Spearman’s Correlation Score. By cBioPortal regression analysis, highly negative relevant coefficients were revealed between Numb and UBE2S (Spearman’s correlation = −0.52, Pearson’s correlation = −0.51) and a moderately negative correlation between Numb and UBE2C (Spearman’s correlation = −0.34, Pearson’s correlation = −0.33) (**Figure 9A**). We also sought to determine the correlation between UBE2S, UBE2C, and Numb expression in three common types of breast cancer, namely ER+, HER2+, and triple-negative breast cancer (TNBC). Consistently, there was a negative correlation between UBE2S and Numb, as well as between UBE2C and Numb, in all three subtypes, with highly negative relevant coefficients indicated in each graph, providing more evidence for the negative correlation between Numb and UBE2S, as well as between Numb and UBE2C (**Figure S4**). To confirm the negative regulation of Numb by UBE2S and UBE2C, we overexpressed (OE) UBE2S or UBE2C in the ER+ cell line MCF7, which exhibits higher Numb expression and lower levels of UBE2S and UBE2C and knocked down UBE2S or UBE2C with small interfering RNA (si) in the ER- cell line MDA-MB-231, which has higher levels of UBE2S and UBE2C and lower Numb expression. It turned out that OE-UBE2S and OE-UBE2C both resulted in decreased Numb expression at the protein and mRNA levels (**Figures 9B, C**, **S5A, C**), whereas a significant increase in Numb was observed by si-UBE2S and si-UBE2C (**Figures 9B, C**, **S5B, D**). To assess tumor cell malignancy, we performed colony formation and Cell Counting Kit-8 (CCK-8) assays. We found MCF7 cells with either UBE2S or UBE2C overexpression demonstrated stronger abilities to form colonies and proliferate, while MDA-MB-231 cells with either UBE2S or UBE2C knockdown showed significantly lower malignancy than control (**Figures 9D, E**). In conclusion, UBE2S and UBE2C both inhibited Numb expression and promoted breast tumor malignancy.

## 4 Discussion

With the increasing incidence of breast cancer all over the world, it is vital to explore the underlying molecular mechanism of this tumor, which jeopardizes women’s lives. Different from other common cancers, breast cancer was divided into four molecular subtypes according to the status of HR, HER-2, and Ki-67, accounting for different therapeutic strategies and prognosis (34). Due to the complexity of clinical manifestations, it is urgent to explore the molecular characteristics of breast cancer, which would facilitate the development of effective clinical interventions. Great progress has been made in the development and progression of breast cancer, but inevitable treatment failure occurs due to the heterogeneity of cancer cells (35–37). The existence of cancer stem cells (CSC) or tumor initiating cells has been put forward and widely validated, accounting for progression, relapse, and treatment failure in multiple cancers (38–40). In this study, we aim to identify a breast cancer cell subpopulation as a potential clinical treatment target marked by possible prognostic markers.

Ubiquitin signaling plays an important role in protein degradation by post-translational modification, involving the multistep enzymatic actions of ubiquitin activating enzyme E1, ubiquitin conjugating enzyme E2, and ubiquitin ligase E3 (41). A growing body of research revealed that abnormalities in ubiquitin signaling were implicated in a variety of malignancies (41–43). As important members of the E2 family, it was reported that UBE2S and UBE2C were highly expressed in cancerous tissues compared with surrounding normal tissues, and aberrant expression of the genes was reported to be involved in tumorigenesis and tumor progression, including breast cancer (11, 44–49). A better understanding of the regulatory mechanisms underlying UBE2S and UBE2C function in breast cancer is expected to identify novel prognostic markers and develop new effective anticancer strategies.

Numb was widely reported as a tumor suppressor in various cancers, and low expression of Numb was related to highly malignant tumor cells (21, 28, 50, 51). As shown in our data, there was reduced expression of Numb at the protein level, suggesting the involvement of ubiquitination and protein degradation (29, 52, 53). Since enzymes are the most attractive targets for drug development, identification of the upstream regulators of Numb downregulation is vital. This is because it has great potential for creating new efficient therapeutic targets in translational medicine. However, less is known concerning the interaction between the ubiquitin conjugating enzymes and Numb.

In the present study, we first proposed that UBE2S and UBE2C confer poor prognosis in breast cancer *via* downregulation of Numb. We reported overexpression of UBE2S and UBE2C and downregulation of Numb in breast cancer compared with normal breast tissue at both mRNA and protein levels. In a paired analysis of breast cancer and adjacent normal tissue, higher levels of UBE2S and UBE2C and lower Numb expression were found in the former. UBE2S and UBE2C also demonstrated increased expression in breast cancer with a more advanced grade and stage, while Numb showed the opposite trend. Since HR+ BC is the most common subtype with a better prognosis than HR− BC, we also found that ER or PR-negative breast cancer tissues or cells had higher levels of UBE2S and UBE2C and lower expression of Numb compared with HR+ breast cancer, providing evidence for the promoting roles of UBE2S and UBE2C and the suppressive role of Numb in breast cancer malignancy. For survival analysis, patients with a higher level of UBE2S or UBE2C had a shorter lifespan than those with a lower expression of UBE2S or UBE2C. Meanwhile, lower Numb expression was accompanied by a worse prognosis in breast cancer patients as well as in ER+ BC patients. Specifically, by combining UBE2S or UBE2C together with Numb, we found patients with low UBE2S or UBE2C and high Numb expression demonstrated a more favorable prognosis. In contrast, patients with high UBE2S or UBE2C and low Numb levels showed a worse outcome. Therefore, we propose that combining UBE2S or UBE2C with Numb may serve as an effective predictor of breast cancer survival.

Mechanically, a notably negative correlation was found between the ubiquitin-conjugating enzymes and Numb through bioinformatic analyses. We further elucidated the underlying mechanisms through gain-and-loss-of-function studies in breast cancer cell lines. It is revealed that overexpression of UBE2S or UBE2C results in decreased Numb expression. However, a significant increase in Numb expression was observed by silencing UBE2S or UBE2C expression in breast cancer cells at both protein and mRNA levels. This confirms the downregulating roles of UBE2S and UBE2C in Numb expression. In addition, we found that overexpression of UBE2S or UBE2C led to increased capabilities of colony formation and cell proliferation while silencing of UBE2S or UBE2C displayed opposite trends in breast cancer cells, indicating the tumor-promoting effect of elevated UBE2S or UBE2C expression.

Taken together, we uncover that UBE2S and UBE2C confer a poor prognosis for breast cancer *via* downregulation of Numb. Breast cancer with higher UBE2S or UBE2C levels and lower Numb expression is correlated with a worse prognosis, providing potential biomarkers for BC therapeutics. It is speculated that UBE2S and UBE2C may work together to regulate Numb expression. However, further validation of the function and regulatory mechanism between UBE2S/UBE2C and Numb is necessary in future work to provide more effective therapeutic interventions.

## Data availability statement

The original contributions presented in the study are included in the article/**Supplementary Material**. Further inquiries can be directed to the corresponding authors.

## Author contributions

XH, XZ, and YG conceptualized and designed the study. YG and XC contributed to the data acquisition and interpretation as well as in methodology and analysis. YG, XZ, and XH were major contributors in writing, review and editing the manuscript. All authors listed have made a substantial, direct, and intellectual contribution to the work and approved it for publication.
