# Supplementary material for: UBE2S and UBE2C confer a poor prognosis to breast cancer via downregulation of Numb
Source: Front Oncol. 2023 Feb 14;13:992233. doi: 10.3389/fonc.2023.992233 (PMC9969189; doi:10.3389/fonc.2023.992233)
Supplement: Supplementary file 1 [file DataSheet_1.docx]

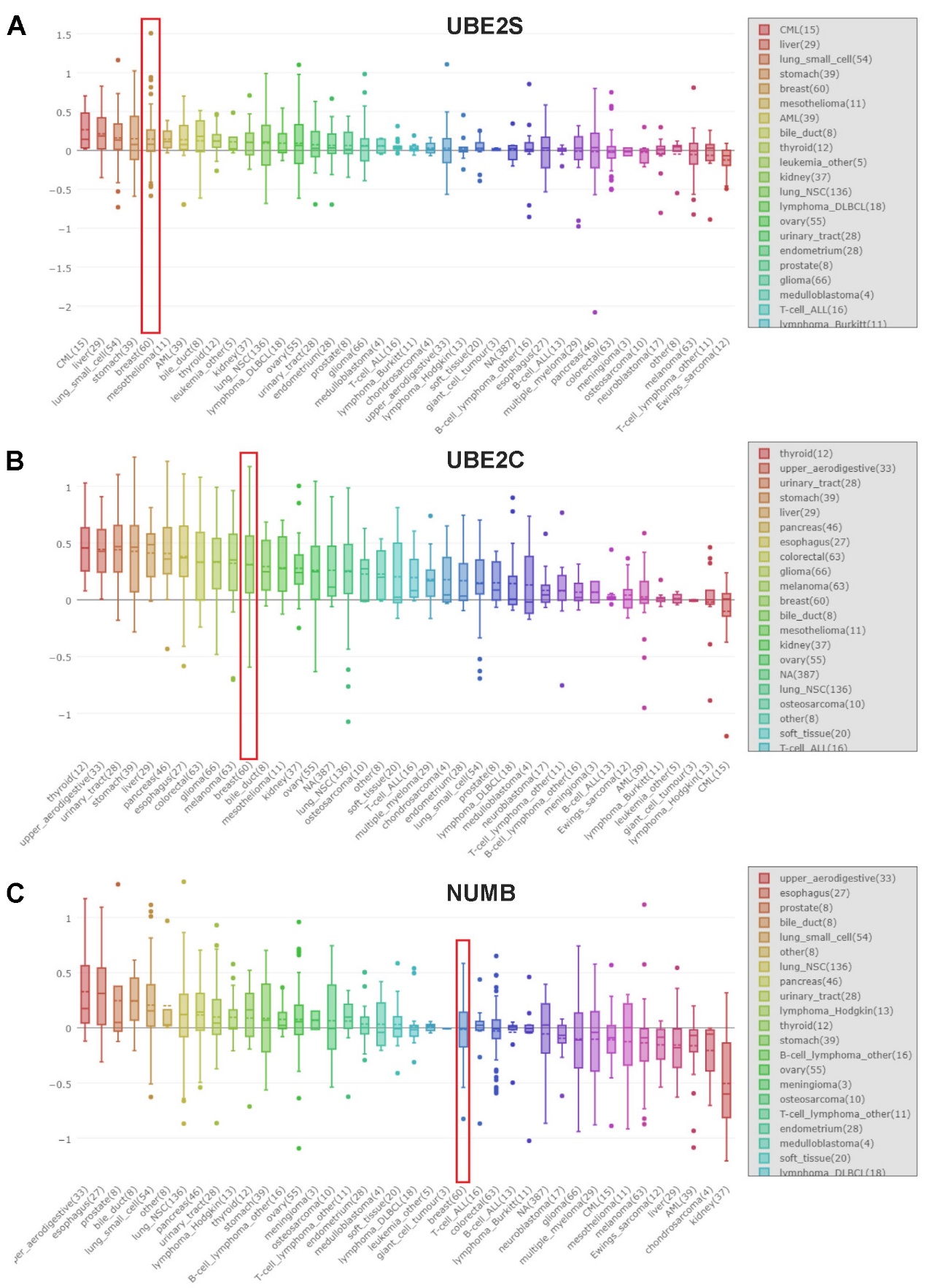


**Figure S1**. **The copy number of UBE2S, UBE2C and Numb in diverse cancer cell lines through CCLE (Cancer Cell Line Encyclopedia) analysis**.

There is significant upregulation of UBE2S and UBE2C copy numbers **(A and B)** while relatively low level of Numb copy number **(C)** in breast cancer cell line (indicated in red frame) compared with other cancer cell lines.


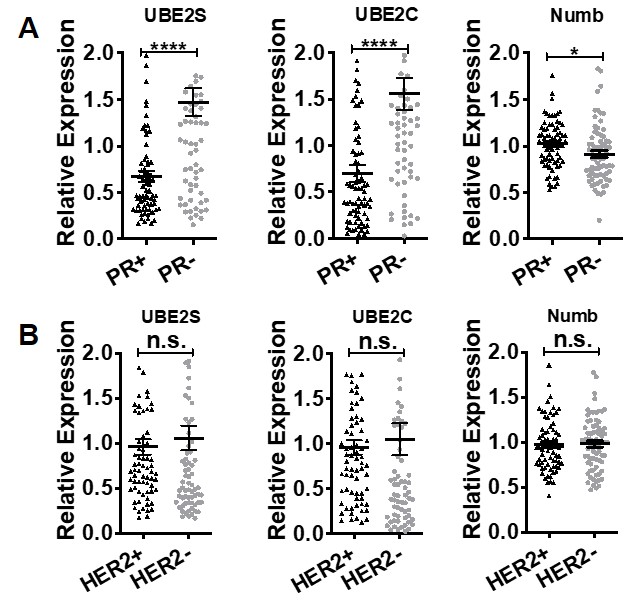


**Figure S2**. **The comparison of UBE2S, UBE2C and Numb expression in breast cancer according to PR and HER2 status (the cBioPortal database)**.

**A**. At mRNA level, UBE2S and UBE2C are upregulated while Numb is decreased in PR negative (PR-) breast cancer tissues compared with PR positive (PR+) group (PR+, n=74, PR-, n=70).

**B**. There are no significant difference in the mRNA levels of UBE2S, UBE2C and Numb between HER2 positive (HER2+) and HER2 negative (HER2-) breast cancer. (HER2+, n=71, HER2-, n=73).

(*t* test was used for the statistical analysis. *, *P* <0.05, **, *P*<0.01, ***, *P*<0.001, ****, *P*<0.0001. n.s., no significance.)

**Figure S3.** **UBE2S, UBE2C and Numb show no correlation with OS or RFS in ER- breast cancer patients by the Kaplan-Meier plotter survival analyses.** The p-value and hazard ratio were indicated in respective graph.


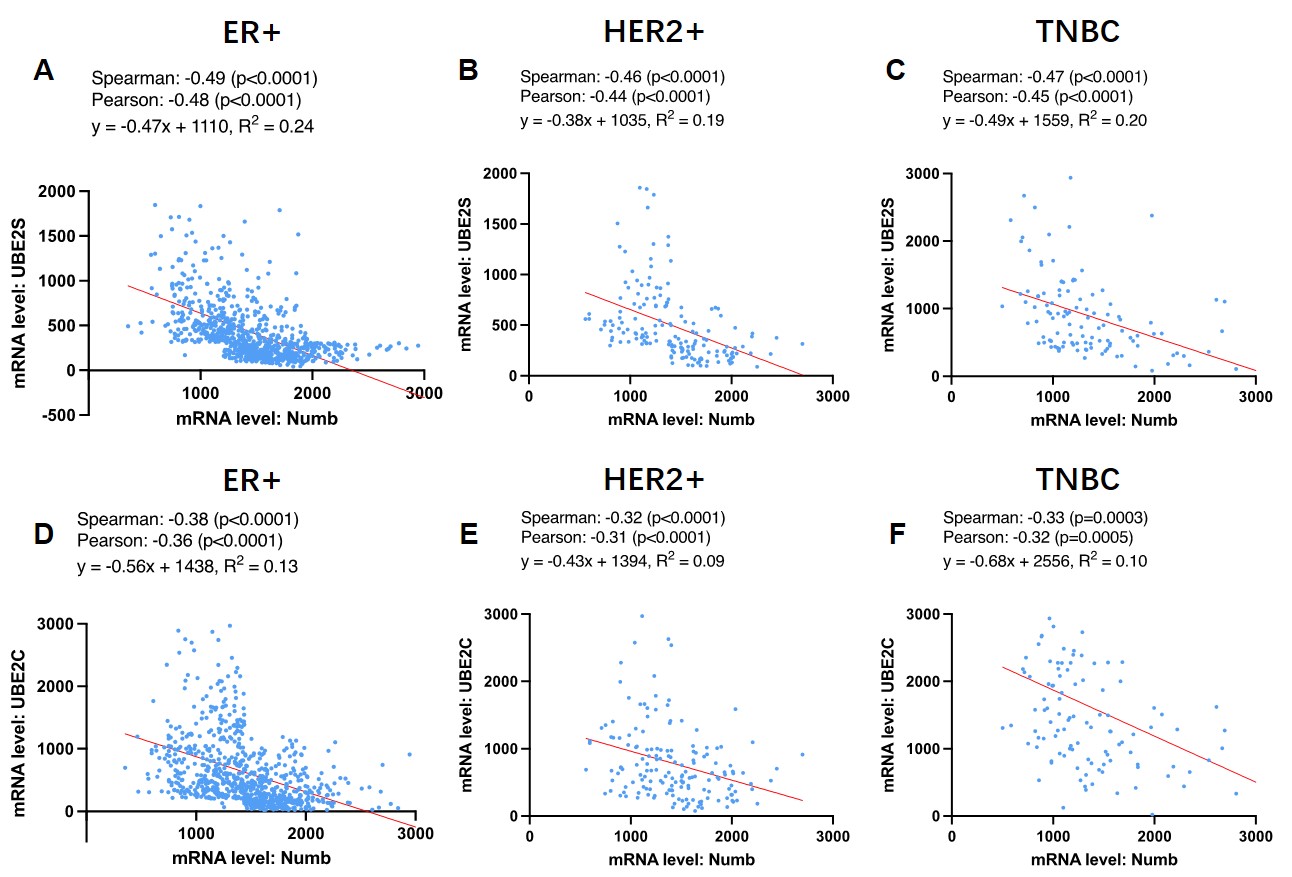
**Figure S4. Numb has a negative correlation with UBE2S (A-C) as well as with UBE2C (D-F) at mRNA level in different subtypes of breast cancer including ER+, HER2+ and TNBC by cBioPortal database analysis.** The Pearson's correlation score, Spearman score and *p*-value were indicated in respective graph.


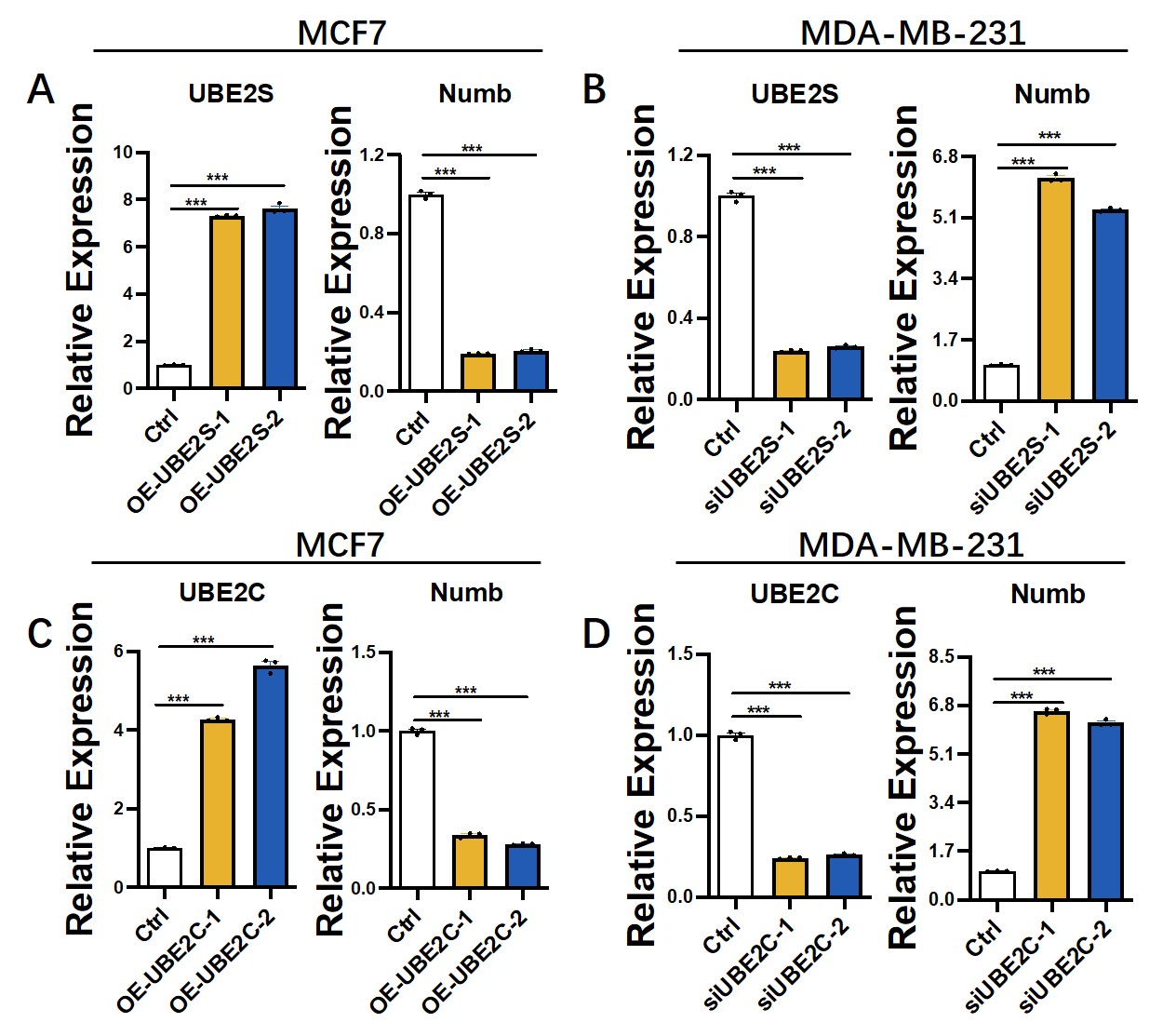


**Figure S5**. **The analysis of UBE2S, UBE2C and Numb in UBE2S or UBE2C overexpressing MCF7 cancer cells as well as in UBE2S or UBE2C downregulating MDA-MB-231 breast cancer cells.**

**A and C**. The overexpression of UBE2S and UBE2C in MCF-7 breast cancer cells transfected with two UBE2S- and UBE2C-overexpressing lentiviruses (OE-UBE2S-1, OE-UBE2S-2, OE-UBE2C-1, OE-UBE2C-2) were confirmed by qRT-PCR (Left). Numb was significantly downregulated in UBE2S or UBE2C overexpressed MCF-7 breast cancer cells compared with the control cells (Ctrl) (Right).

**B and D**. Two small interfering RNA of UBE2S and UBE2C were applied transfecting MDA-MB-231 breast cancer cells (siUBE2S-1, siUBE2S-2, siUBE2C-1, siUBE2C-2) and the transfection efficiencies were confirmed by qRT-PCR (Left). There was a notably upregulation of Numb expression in UBE2S or UBE2C downregulated MDA-MB-231 cancer cells compared with the control cells (Ctrl) (Right).

(*t* test was used for the statistical analysis. *, *P* <0.05, **, *P*<0.01, ***, *P*<0.001, ****, *P*<0.0001. n.s., no significance.
