# Supplementary material for: UBE2S and UBE2C confer a poor prognosis to breast cancer via downregulation of Numb
Source: Front Oncol. 2023 Feb 14;13:992233. doi: 10.3389/fonc.2023.992233 (PMC9969189; doi:10.3389/fonc.2023.992233)
Supplement: Supplementary file 2 [file Table_1.docx]

**Supplementary Table 1. Univariate regression analyses of overall survival (OS) and regression-free survival (RFS) using Cox proportional hazards model.**

| **Variables** | **OS** | | **RFS** | |
| --- | --- | --- | --- | --- |
|  | **HR (95% CI)** | ***P*** | **HR (95% CI)** | ***P*** |
| **Expression** |  |  |  |  |
| **Others** | Reference | - | Reference | - |
| **UBE2S low + NUMB high** | 0.42 (0.24-0.76) | 0.004 | 0.30 (0.11-0.84) | 0.022 |
| **UBE2S high + NUMB low** | 1.78 (1.09-2.89) | 0.021 | 2.46 (1.15-5.24) | 0.020 |
|  |  |  |  |  |
| **Expression** |  |  |  |  |
| **Others** | Reference | - | Reference | - |
| **UBE2C low + NUMB high** | 0.22 (0.13-0.38) | < 0.001 | 0.28 (0.11-0.71) | 0.007 |
| **UBE2C high + NUMB low** | 2.17 (1.37-3.45) | 0.001 | 3.53 (1.65-7.57) | 0.001 |
